# *Echinococcus multilocularis* in Urban Coyotes, Alberta, Canada

**DOI:** 10.3201/eid.1810.120119

**Published:** 2012-10

**Authors:** Stefano Catalano, Manigandan Lejeune, Stefano Liccioli, Guilherme G. Verocai, Karen M. Gesy, Emily J. Jenkins, Susan J. Kutz, Carmen Fuentealba, Padraig J. Duignan, Alessandro Massolo

**Affiliations:** University of Calgary, Calgary, Alberta, Canada (S. Catalano, M. Lejeune, S. Liccioli, G.G. Verocai, S.J. Kutz, P.J. Duignan, A. Massolo);; University of Saskatchewan, Saskatoon, Saskatchewan, Canada (K.M. Gesy, E.J. Jenkins);; and Ross University, Basseterre, Saint Kitts, Saint Kitts, and Nevis (C. Fuentealba)

**Keywords:** Echinococcus multilocularis, alveolar echinococcosis, coyotes, Canis latrans, Alberta, Canada, parasites, cestodes, zoonoses

## Abstract

*Echinococcus multilocularis* is a zoonotic parasite in wild canids. We determined its frequency in urban coyotes (*Canis latrans*) in Alberta, Canada. We detected *E. multilocularis* in 23 of 91 coyotes in this region. This parasite is a public health concern throughout the Northern Hemisphere, partly because of increased urbanization of wild canids.

*Echinococcus multilocularis* is the causative agent of alveolar echinococcosis in humans. This disease is a serious problem because it requires costly long-term therapy, has high case-fatality rate, and is increasing in incidence in Europe (*1*). This parasitic cestode has a predominantly wild animal cycle involving foxes (*Vulpes* spp.) and other wild canids, including coyotes (*Canis latrans*), as definitive hosts. However, it can also establish an anthropogenic life cycle in which dogs and cats are the final hosts. Rodents are the primary intermediate hosts in which the alveolar/multivesicular hydatid cysts grow and are often fatal. Humans are aberrant intermediate hosts for *E. multilocularis* (*2*).

In North America, *E. multilocularis* was believed to be restricted to the northern tundra zone of Alaska, USA, and Canada until it was reported in red foxes (*Vulpes vulpes*) from North Dakota, USA (*3*). This parasite has now been reported in the southern half of 3 provinces in Canada (Manitoba, Saskatchewan, and Alberta) and in 13 contiguous states in the United States (*1*).

Foxes are the traditional definitive hosts for *E. multilocularis* worldwide. However, in North America, coyotes may be prominent hosts, particularly when they are more abundant than foxes. *E. multilocularis* was reported in 7 (4.1%) of 171 coyotes in the northcentral United States in the late 1960s (*3*), and subsequently prevalences ranging from 19.0% to 35.0% have been reported in coyotes in the central United States (*4*).

In Canada, *E. multilocularis* was detected in 10 (23.0%) of 43 coyotes in Riding Mountain National Park, Manitoba (*5*). In Alberta, 1 case was recorded from the aspen parkland in 1973 (*5*) but it was not found in coyotes from forested regions and southern prairies (*6*,*7*). Nonetheless, *E. multilocularis* is generally considered enzootic to central and southern Alberta on the basis of its prevalence in rodent intermediate hosts. During the 1970s, sixty-three (22.3%) of 283 deer mice (*Peromyscus maniculatus*) trapped in periurban areas of Edmonton were positive for alveolar hydatid cysts (*8*), and *E. multilocularis* was also detected in 2 deer mice collected <1.8 km from Lethbridge in southern Alberta (*9*).

Because mice and voles (family Cricetidae, including *Peromyscus* spp.) have been reported as main prey (70.1%) of coyotes in Calgary (*10*), and coyotes are common in urban areas of Calgary and Edmonton, we suspected a role for this carnivore in the maintenance of the wild animal cycle of *E. multilocularis* in such urban settings. Thus, we aimed to ascertain the frequency of *E. multilocularis* in coyotes from metropolitan areas in Alberta, Canada.

## The Study

**Figure 1:**
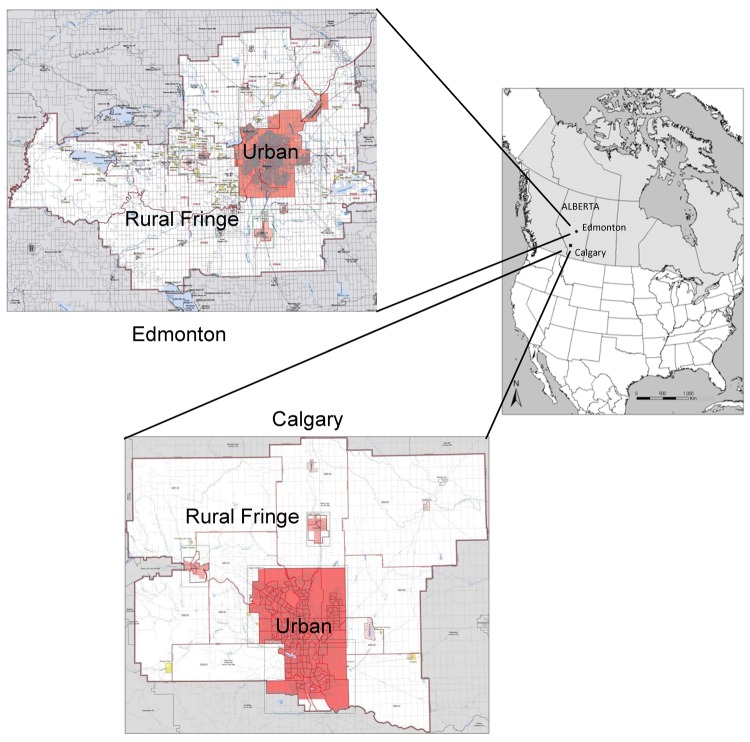
Calgary and Edmonton, Alberta, Canada, census metropolitan areas in which 91 coyote carcasses were collected during 2009–2011 and tested for *Echinococcus multilocularis*. Reference maps (2006) were obtained from the Geography Division, Statistics Canada (www12.statcan.gc.ca/census-recensement/2006/geo/index-eng.cfm). Urban core areas and surrounding rural fringes are indicated. For Edmonton, 5 (62.5%) of 8 carcasses were positive. For Calgary, 18 (20.5%) of 83 carcasses were positive: 9 (27.3%) of 33 from the rural fringe, 4 (14.8%) of 27 from the urban area, and 5 (21.7%) of 23 whose locations of collection were not accurate enough to be classified as urban or from the rural fringe.

Ninety-one hunted or road-killed coyotes were collected during October 2009–July 2011. Most (n = 83) of the carcasses were from the Calgary census metropolitan area (CMA) (Figure 1). The remainder (n = 8) were opportunistically collected from the Edmonton CMA. Of those from the Calgary CMA, the exact location of collection was known for 60 animals: 27 were from Calgary and 33 were from the rural fringe, including 2 near Strathmore. Of the carcasses from the Edmonton CMA, 7 were from Edmonton and 1 was from a periurban site. Sex and age of 90 of the coyotes were recorded.

Before necropsy, all carcasses were stored at −20°C. Gastrointestinal tracts collected at necropsy were refrozen at −80°C for 3–5 days to inactivate *Echinococcus* spp. eggs. Once thawed and dissected, intestinal contents were washed, cleared of debris, and passed through a sieve (500-µm pores), and the material in the sieve was examined for *Echinococcus* spp.

**Figure 2:**
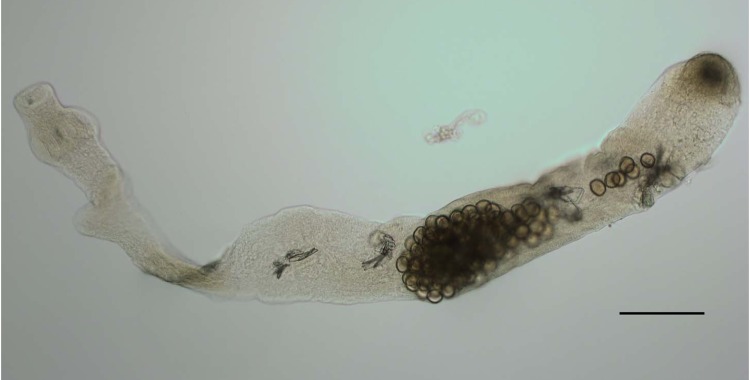
Differential interference contrast micrograph of a representative *Echinococcus multilocularis* isolate from a coyote carcass in Alberta, Canada, October 2009–July 2011. The parasite was 2,059.72 μm long, as measured by using an Olympus BX53 microscope and software (http://microscope.olympus-global.com/en/ga/product/bx53/sf04.cfm). Scale bar = 200 μm.

Adult tapeworms were counted and identified as *E. multilocularis* on the basis of morphologic features (Figure 2). To confirm morphologic identification, PCR was performed by using species-specific primers (*11*). Briefly, a representative adult worm from each positive animal was lysed in 50 μL of DNA extraction buffer (500 mmol/L KCl, 100 mmol/L Tris-HCl, pH 8.3, 15 mmol/L MgCl_2_, 10 mol/L dithiothreitol, and 4.5% Tween 20) containing 2 μL of proteinase K. This lysate was further diluted (1:20 in double-distilled water), and 2 μL was used for PCR. Amplicons of an expected 395 bp confirmed infection with *E. multilocularis*.

*E. multilocularis* was identified in 23 (25.3%) of 91 coyotes by using morphologic and molecular identification. Among positive animals, 18 (20.5%) of 83 were from the Calgary CMA and 5 (62.5%) of 8 were from the Edmonton CMA. In the Calgary CMA, 4 (14.8%) of 27 positive animals were found in the city and 9 (27.3%) of 33 were found in the rural fringe (Figure 1). Five (21.73%) of 23 coyotes for which the location was not recorded were also positive.

*E. multilocularis* intensity (number of cestodes per host) ranged from 1 to 1,400 (median 20.5). The frequency of infection was significantly higher in male coyotes (n = 44, 34.19%) than in female coyotes (n = 46, 15.2%; χ^2^ 4.337, df 1, *P_exact_* = 0.05) (Table). No difference was detected between 43 juvenile coyotes and 47 adult coyotes (Table).

**Table:** *Echinococcus multilocularis* in coyotes carcasses collected in Calgary (n = 83) and Edmonton (n = 8) census metropolitan areas, Alberta, Canada, October 2009–July 2011*

Characteristic	Total	No. (%) positive or median (range)	No. negative	IQ distance	χ^2^ (z)	df	p_exact_ value†
Sex‡							
M	44	15 (34.1)	29				
F	46	7 (15.2)	39	NA	**4.337**	1	**0.05**
Parasite intensity							
M	NA	9 (1–1,400)	NA	83			
F	NA	59 (9–822)	NA	137	(−1.406)		0.19
Age‡							
Juvenile	43	14 (33.3)	29	NA			
Adult	47	8 (17.0)	39	NA	1.661	1	0.226
Parasite intensity							
Juvenile	NA	9 (1–151)	NA	71			
Adult	NA	32 (1–1,400)	NA	520	(−0.737)		0.518

*Values in **boldface** indicate a significant difference. Higher prevalence in male coyotes suggests a role for sex in parasite dispersion. Frequencies of cestodes in males vs. females and juveniles vs. adults were analyzed by using χ^2^ test. Parasite intensity (no. parasites per host) among sex and age classes was compared by using Mann-Whitney test for independent samples. IQ, interquartile distance; NA, not applicable. †Probability of distribution was estimated by using the permutation approach (p_exact_). ‡Sex and age of 1 coyote were not recorded.

## Conclusions

We demonstrated that *E. multilocularis* is common in coyotes of metropolitan areas of Calgary and Edmonton, Alberta, Canada, including their urban cores. This finding might indicate an emerging phenomenon similar to that observed in Europe with infiltration of urban centers by *E. multilocularis* caused by an increase in red foxes in cities such as Copenhagen, Geneva, and Zurich (*2*). In Alberta, this phenomenon may be caused by coyotes occupying the urban landscape or by city sprawl invading the natural habitats of coyotes.

Our data suggest that *E. multilocularis* is enzootic in coyotes in Alberta and that perpetuation of the wild animal cycle of *E. multilocularis* within cities and surroundings and potential infection of domestic dogs may pose a zoonotic risk, as documented on Saint Lawrence Island, Alaska, and in China (*2*,*12*). With a considerable increase in domestic dog population of Calgary (32.1% increase since 2001, a total of 122,325 dogs in 2010; Animal and Bylaw Services Survey 2010, www.calgary.ca/CSPS/ABS/Pages/home.aspx) and substantial human population growth (32.9% increase in Calgary since 1999; Statistics Canada, 2009, www.statcan.gc.ca/start-debut-eng.html), awareness is needed of potential transmission risks associated with changing city landscapes and *E. multilocularis* in the urban environment.

In Canada, only 1 autochthonous human case of alveolar echinococcosis has been reported in Manitoba (*13*). However, imported cases have been described. In Alberta, there are no known reports of alveolar echinococcosis. This finding may be caused by the long incubation time required for clinical manifestation in humans (*12*) or a strain of *E. multilocularis* with a low zoonotic potential. Although there is little evidence of human risk from the strain of *E. multilocularis* in central North America (*14*), a human case caused by this strain has been confirmed (*15*).

Our finding of *E. multilocularis* in coyotes in urban regions in Alberta suggests that surveillance for this parasite should be increased in North America. Although removal of this parasite from domestic dogs and cats is effective, eradication from free-ranging definitive hosts may be unfeasible (*2*,*12*). Interventions other than improving public awareness about prevention and transmission risk are probably unnecessary, and public health messages should target veterinarians and dog owners because domestic dogs probably represent the main infection route for humans in North America (*2*,*12*). Genetic characterization of *E. multilocularis* and spatially explicit transmission models should also be developed to better assess risks of this emerging zoonosis in North America and worldwide.
